# Case Report: An Unusual Presentation of Cardiovascular Involvement in Eosinophilic Granulomatosis With Polyangiitis

**DOI:** 10.3389/fcvm.2022.928192

**Published:** 2022-06-28

**Authors:** Yajuan Li, Hui Zhou, Yaou Zhou, Haixiong Tang

**Affiliations:** ^1^Department of Radiology, Xiangya Hospital Central South University, Changsha, China; ^2^National Clinical Research Center for Geriatric Disorders, Xiangya Hospital Central South University, Changsha, China; ^3^Department of Rheumatology and Immunology, Xiangya Hospital Central South University, Changsha, China; ^4^Department of Radiology, The Fourth People's Hospital of Chenzhou, Chenzhou, China

**Keywords:** eosinophilic granulomatosis with polyangiitis, Löffler endocarditis, thrombosis, pulmonary hypertension, right-sided heart failure, cardiac magnetic resonance imaging

## Abstract

**Background:**

Because eosinophilic granulomatosis with polyangiitis (EGPA) is so rare and the symptoms so varied, it can be a challenge to get a correct diagnosis in clinical practice. Cardiovascular involvement is the main cause of death of EGPA. We are the first to report of cardiac magnetic resonance (CMR) findings about right-sided heart involvement in EGPA.

**Patient Findings:**

The initial abnormalities detected by CMR were Löffler endocarditis with extensive thrombosis and left ventricular (LV) dysfunction. After active treatment, LV systolic function recovered and endocarditis with thrombosis significantly improved, but there was rapidly progressive pulmonary hypertension, enlargement of right atrium and right ventricle and persistent right-sided heart failure. The patient eventually died of sudden cardiac death 6 months after hospital discharge.

**Conclusions:**

Löffler endocarditis and right-sided heart involvement are both rare presentations in patients with EGPA. CMR is a reliable non-invasive tool to precisely and comprehensively assess cardiovascular involvement in EGPA.

## Introduction

Eosinophilic granulomatosis with polyangiitis (EGPA) is a systemic necrotizing vasculitis of small- and medium-size vessels, characterized by extravascular granulomas, eosinophilia, and tissue infiltration by eosinophils of multiple organs, including heart, lungs, skin, gastrointestinal tract, kidneys, and peripheral nerves (1). Cardiac involvement in EGPA is associated with a poor prognosis and high mortality. Therefore, early diagnosis and treatment are essential to prevent the acceleration of cardiac involvement in patients with EGPA (1, 2). We describe a rare case of EGPA characterized by involvement of heart and pulmonary artery.

## Case Presentation

**Figure 1:**
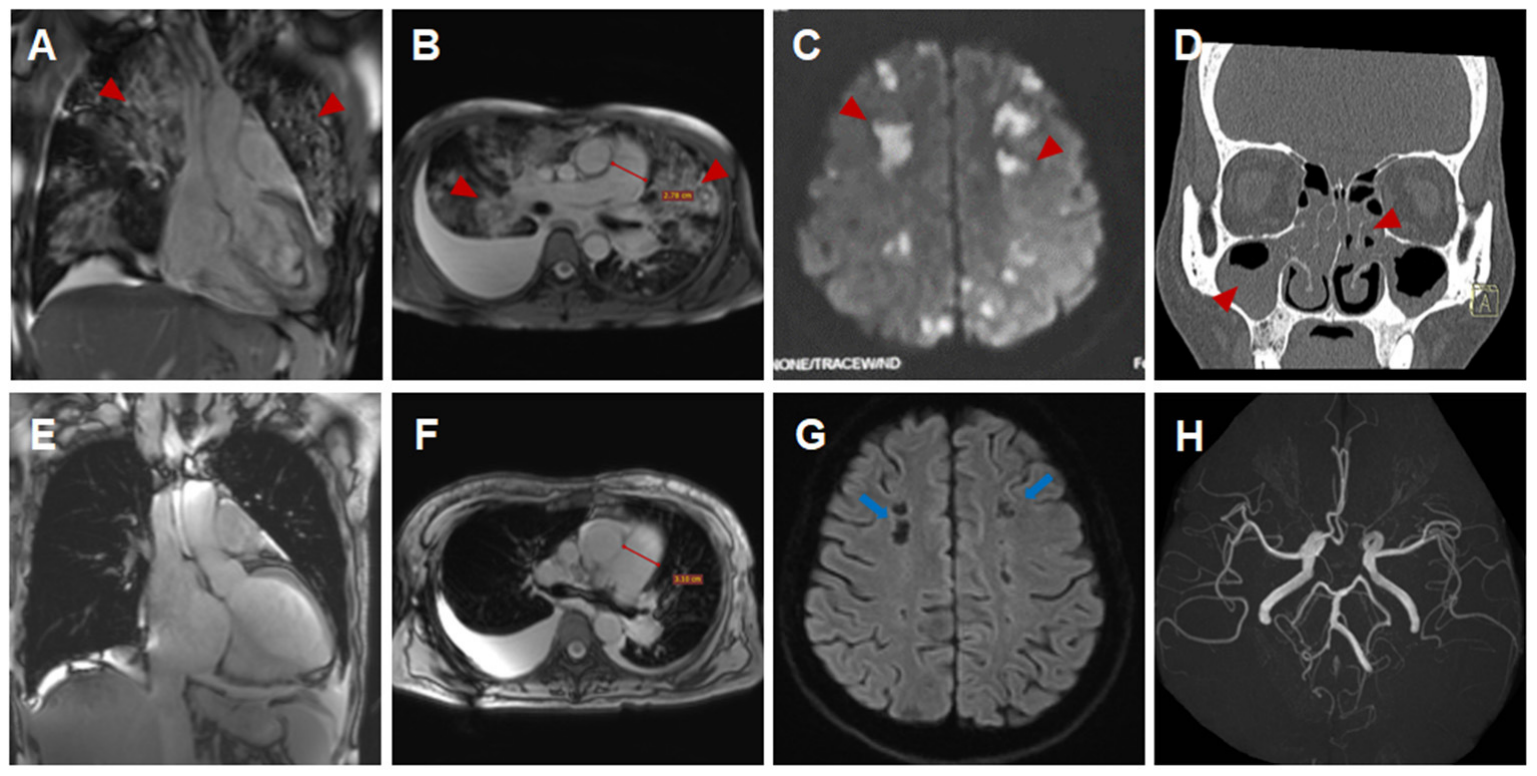
Imaging findings of eosinophilic granulomatosis with polyangiitis **(A–D,H)** Imaging findings during the initial hospitalization (**A,D**, coronal view; **B,C,H**, transverse view). Multiple patchy consolidations of both lungs (**A,B**, triangular arrows) on CMR images, multiple acute cerebral infarctions (**C**, triangular arrows) with no obvious cerebrovascular stenosis (**H**, triangular arrows) on brain MR images and sinusitis and nasal polyps (**D**, triangular arrows) on reconstructed CT images. **(E–G)** Imaging findings during the subsequent hospitalization (**E**, coronal view; **F,G**, transverse view). Significantly absorbed multiple lesions of both lungs (**E,F**), the pulmonary hypertension with increased diameters of pulmonary trunk from 28 mm **(B)** to 32 mm **(F)** and multiple encephalomalacia (**G**, long arrows).

**Figure 2:**
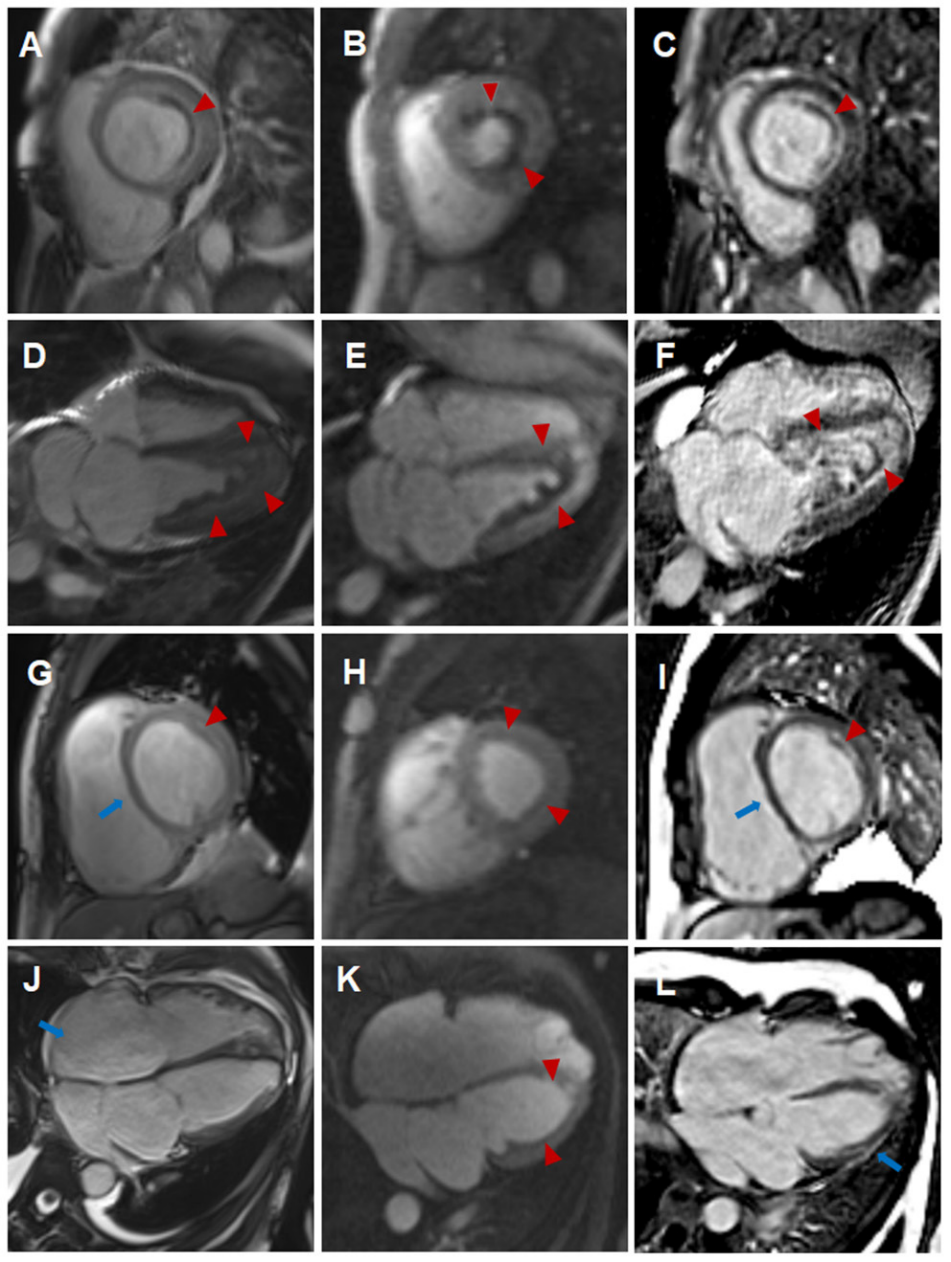
Comparison of cardiovascular involvement detected by CMR during two separate hospitalizations **(A–F)** CMR findings during the initial hospitalization (**A–C**, short-axis view; **D–F**, four-chamber view). Distinct high-signal-intensity plane separating the thrombus from underlying myocardium on end-diastolic cine images (**A,D**, triangular arrows), endocardial surface hypoperfusion zone on perfusion images (**B,E**, triangular arrows) and endocardial enhancement and overlying non-enhancing thrombus on LGE images (**C,F**, triangular arrows). **(G–L)** CMR findings during the subsequent hospitalization (**G–I**, short-axis view; **J–L**, four-chamber view). Enlargement of right atrium and right ventricle, flat ventricular septum (**G,J**, long arrows) and significantly reduced high-signal-intensity plane and thrombus (**G,J**, triangular arrows) on cine images, significantly reduced endocardial surface hypoperfusion zone on perfusion images (**H,K**, triangular arrows) and significantly thinning endocardial enhancement and overlying thrombus (**I,L**, triangular arrows), new strip-like LGE in the lateral wall (**L**, long arrows) on LGE images, representing replacement fibrosis.

A 44-year-old female patient presented to our hospital with a 1-month history of dizziness, fatigue, chest tightness, and shortness of breath. She also had ecchymosis of the lower legs. The patient presented with a 10-year history of asthma, sinusitis, and nasal polyps (Figure 1D). Electrocardiograph (ECG) demonstrated QS waves in leads V2-V3, ST segment depression in leads V5-V6, and T-wave inversion. Blood pressure was 90/62 mmHg, pulse was 112 beats/minute. Laboratory findings (Table 1) showed increase in the percentage of eosinophils (47.8%), erythrocyte sedimentation rate (ESR) (32 mm/H), C-Reactive protein (CRP) (24.70 mg/L), N-terminal pro-B-type natriuretic peptide (NT-proBNP) (12,045 pg/mL), and troponin I (1.760 ng/mL). Diagnostic work up revealed negative anti-nuclear antibodies (ANA), ANCA, anti-MPO antibodies, and rheumatoid factor. The patient's renal function was normal and there was no evidence of blood infection. Diffusion-weighted imaging of the brain magnetic resonance imaging (MRI) revealed multiple acute cerebral infarctions (Figure 1C) with no obvious cerebrovascular stenosis on magnetic resonance angiography (MRA) (Figure 1H). Chest CT and Cardiac magnetic resonance (CMR) localizing images showed multiple patchy consolidations of both lungs and bilateral pleural effusion (Figures 1A,B). After completing a series of examinations and multi-disciplinary discussions, the patient met the inclusion criteria of the American College of Rheumatology 1990 criteria for the classification of EGPA (3). Transthoracic echocardiography showed numerous deposits in the mural left ventricular (LV) endocardium, obliteration of the apical portion of the left ventricle, enlarged left atrium, moderate pulmonary hypertension [the estimated systolic pulmonary artery pressure (eSPAP) was 46 mmHg] and a small pericardial effusion. ^18^F-FDG PET-CT showed accumulation of FDG in the lungs and myocardium. CMR was used to assess the cardiovascular involvement which was performed on a 3.0 T MRI at 8 days after admission, confirming Löffler endocarditis (endocardial thickening, edema and enhancement) with LV thrombus on cine, perfusion, and LGE images (Figures 2A–F, Supplementary Videos 1–6), LV volume enlargement and mildly reduced LV systolic function, normal right ventricular volume, and function (Table 2, Supplementary Videos 1–4).

**Table 1:** Laboratory findings during two separate hospitalizations.

	**The percentage of eosinophils**	**ESR**	**CRP**	**NT-proBNP**	**Troponin I**
The initial admission	47.8%	32 mm/H	24.70 mg/L	12,045.00 pg/mL	1.760 ng/mL
The initial discharge	1.0%	111 mm/H	64.30 mg/L	4,072.07 pg/mL	0.020 ng/mL
The subsequent admission	1.1%	5 mm/H	1.72 mg/L	4,472.00 pg/mL	0.020 ng/mL
The subsequent discharge	0.7%	7 mm/H	5.11 mg/L	5,451.00 pg/mL	0.030 ng/mL
Reference range	0.4–8.0%	0–26 mm/H	0–8.00 mg/L	<125 pg/mL	<0.040 ng/mL

*ESR, erythrocyte sedimentation rate; CRP, C-Reactive protein; NT-proBNP, N-terminal pro-B-type natriuretic peptide*.

**Table 2:** Comparison of CMR results during two separate hospitalizations.

	**The initial CMR**	**The subsequent CMR**	**Reference range**
LVEF (%)	46	60	57–81
LVEDVI (mL/m2)	70	75	51–95
LVESVI (mL/m2)	38	27	11–35
RVEF (%)	53	24	50–78
RVEDVI (mL/m2)	62	137	42–118
RVESVI (mL/m2)	29	105	6–54
LA (cm2)	21	22	<24
RA (cm2)	15	32	<23

*LVEF, left ventricular ejection fraction; LVEDVI, left ventricular end-diastolic volume indexed; LVESVI, left ventricular end-systolic volume indexed; RVEF, right ventricular ejection fraction; RVEDVI, right ventricular end-diastolic volume indexed; RVESVI, right ventricular end-systolic volume indexed; LA, left atrium; RA, right atrium*.

Eosinophils reduced to normal (Table 1) and her symptoms gradually improved after treatment according to the 2015 EGPA Consensus Task Force recommendations (4). Chest CT showed that the diffuse lesions of both lungs were absorbed, and the pleural effusion was reduced after the treatment. Unfortunately, the patient was readmitted for dyspnea, bilateral lower limb edema and systemic ecchymosis after 10 months. Transthoracic echocardiography showed deposits in the mural LV endocardium were significantly reduced, the LV diameter returned to normal but greater diameter of right atrium (RA) and right ventricle (RV) with moderate-to-severe regurgitation of tricuspid valve and pulmonary valve, severe pulmonary hypertension (eSPAP was 71 mmHg). CMR imaging was performed again, showing enlargement of RA and RV (Table 2, Figure 2J, Supplementary Videos 9–12), significantly improved endocardial enhancement and overlying thrombus on cine, perfusion and LGE images (Figures 2G–L, Supplementary Videos 7–12), remarkably reduced RV systolic function but improvement of LV systolic function (Table 2, Supplementary Videos 7–10), new strip-like LGE in the lateral wall (Figure 2L), representing replacement fibrosis and increased diameters of pulmonary trunk from 28 mm to 32 mm (Figures 1B,F). Multiple lesions of both lungs were significantly absorbed (Figures 1E,F) and encephalomalacia were formed (Figure 1G). Despite the patient received intensive immunosuppressive treatment combing glucocorticoid and immunosuppressant (cyclophosphamide) and aggressive anti-heart failure therapy, the symptoms of right-sided heart failure were not completely relieved and sudden cardiac death occurred 6 months after hospital discharge.

## Discussion

Although EGPA belongs to the spectrum of ANCA-associated vasculitis, <50% of patients with EGPA are ANCA positive (1). Cardiac involvement occurs in approximately 15–60% of EGPA patients and can present as pericarditis, myocarditis, acute heart failure, acute myocardial infarction, valvular heart disease, and especially those who are ANCA negative (1, 2, 5). Löffler endocarditis is present in 50–60% hypereosinophilic syndrome (HES), but rarely reported in patient with EGPA (6). Through the literature search, there is no report of EGPA patient involving the right-sided heart so far.

For the evaluation of cardiac involvements, the unique advantage of CMR lies in the comprehensive evaluation of cardiac volume, function, and myocardial tissue characterization. The initial abnormalities of this case detected by CMR were Löffler endocarditis with extensive thrombosis in LV and LV dysfunction. After treatment, pulmonary lesions and endocarditis with thrombosis were significantly improved, with LV systolic function recovered, but pulmonary hypertension rapidly progressed and right-sided heart enlargement and right-sided heart failure emerged. Pilania et al. (7) also reported that pulmonary arterial hypertension is a rare occurrence in ANCA-associated vasculitis. Löffler endocarditis of HES or EGPA progresses through three stages, namely acute necrotic stage (infiltration of eosinophils in the myocardium), thrombotic stage (mural thrombi formation along the damaged endocardium), and fibrotic stage (the granulation tissue changing into hyaline fibrosis), which may overlap (2, 8). Myocardial fibrosis may develop rapidly and immediate aggressive treatment may help to slow the progression of chronic heart failure (8–10). Right-sided heart failure can be a consequence of left ventricular dysfunction; however, Fitchett et al. (11) believed that right ventricular failure might in certain circumstances develop either before or after left ventricular failure when there is a diffuse myocardial disease. Therefore, we hypothesize that emerging right-sided heart failure of this patient could be induced by the combined effect of progressive and refractory pulmonary hypertension caused by pulmonary vasculitis and the diffuse myocardial damage. T1 and T2 mappings are new parametric quantitative sequences, which provide tissue-specific T1 and T2 values. Puntmann et al. (12) believed that T1 and T2 mappings may support non-invasive recognition of cardiac involvement and activity of myocardial inflammation. Cereda et al. (13) reported that CMR parameters of interstitial fibrosis including native T1 and extracellular volume fraction (ECV) were significantly more elevated in patients with EGPA in clinical remission compared to healthy subjects. Lagan et al. (14) also found that stable EGPA was associated with focal replacement (non-ischemic LGE) and diffuse interstitial myocardial fibrosis (elevated native T1 and ECV), but myocardial T2 and capillary permeability were no different in EGPA compared to control. Since the initial scanning machine was not equipped with mapping sequences, T1 and T2 mappings were underwent during the subsequent hospitalization and elevated native T1, ECV and normal T2 were found, which was consistent with the results of previous studies (13, 14).

Because EGPA is so rare and the symptoms so varied, it can be a challenge to get a correct diagnosis in clinical practice. EGPA with <50% ANCA positive can also easily be misdiagnosed as HES due to incomplete history collection. However, chief manifestations of cardiovascular involvement and therapeutic schedule of HES and EGPA are quite different (15). EGPA patients often present with pericarditis, myocarditis, valvular heart disease, restrictive cardiomyopathy and acute myocardial infarction from vasculitic pathology of coronary artery, while Löffler endocarditis is the common cardiac performance of HES. Corticosteroid therapy remains first drug of choice in both HES and EGPA, immunosuppressant (e.g., cyclophosphamide) should be added in EGPA patients with cardiac involvement.

There are still some limitations of this study. The histological examination of tissue biopsies of the lungs and the heart cannot be performed due to the high risk of thrombus shedding and more cases are needed to further validate our findings.

## Conclusion

Right-sided heart involvement is extremely rare in EGPA, but may be associated with a poor prognosis. CMR is a reliable non-invasive way to precisely and comprehensively assess cardiac volume, function, and myocardial tissue characterization. In this regard, we highly recommend CMR as initial and follow-up diagnostic tool to evaluate cardiovascular involvements for all the patients with EGPA.

## Data Availability Statement

The original contributions presented in the study are included in the article/Supplementary Material, further inquiries can be directed to the corresponding author.

## Ethics Statement

The studies involving human participants were reviewed and approved by the Ethics Committee of the Xiangya Hospital of Central South University. The patients/participants provided their written informed consent to participate in this study. Written informed consent was obtained from the individual(s) for the publication of any potentially identifiable images or data included in this article.

## Author Contributions

All authors listed have made a substantial, direct, and intellectual contribution to the work and approved it for publication.

## Funding

The Natural Science Foundation of Hunan Province (2021JJ31131), China.

## Conflict of Interest

The authors declare that the research was conducted in the absence of any commercial or financial relationships that could be construed as a potential conflict of interest.

## Publisher's Note

All claims expressed in this article are solely those of the authors and do not necessarily represent those of their affiliated organizations, or those of the publisher, the editors and the reviewers. Any product that may be evaluated in this article, or claim that may be made by its manufacturer, is not guaranteed or endorsed by the publisher.
