# Supplementary material for: Case Report: An Unusual Presentation of Cardiovascular Involvement in Eosinophilic Granulomatosis With Polyangiitis
Source: Front Cardiovasc Med. 2022 Jun 28;9:928192. doi: 10.3389/fcvm.2022.928192 (PMC9273878; doi:10.3389/fcvm.2022.928192)
Supplement: Supplementary file 13 [file Data_Sheet_1.DOCX]

**Videos Legends**

**(Supplementary Videos 1-6) CMR findings during the initial hospitalization.**

LV volume enlargement and mildly reduced LV systolic function, normal right ventricular volume and function, and distinct high-signal-intensity plane separating the thrombus from underlying myocardium on end-diastolic cine images (**Videos 1-4**, two-chamber, three-chamber, four-chamber and short-axis view of cine images, respectively). Endocardial surface diffuse hypoperfusion on perfusion images (**Videos 5-6**, four-chamber and short-axis view of perfusion images, respectively).

**(Supplementary Videos 7-12) CMR findings during the subsequent hospitalization.**

Enlargement of right atrium and right ventricle, flat ventricular septum, significantly improved endocardial enhancement and overlying thrombus, remarkably reduced RV systolic function but improvement of LV systolic function ( **Videos 7-10**, two-chamber, three-chamber, four-chamber and short-axis view of cine images, respectively). Significantly reduced endocardial surface hypoperfusion zone on perfusion images (**Videos 11-12**, four-chamber and short-axis view of perfusion images, respectively).
